# Ballodiolic Acid A and B: Two New ROS, (^•^OH), (ONOO^−^) Scavenging and Potent Antimicrobial Constituents Isolated from *Ballota pseudodictamnus* (L.) Benth.

**DOI:** 10.3390/pharmaceutics13030402

**Published:** 2021-03-17

**Authors:** Fozia, Asmat Shaheen, Ijaz Ahmad, Syed Badar Amin, Nisar Ahmad, Riaz Ullah, Ahmed Bari, Muhammad Sohaib, Hafiz Majid Mahmood, Abdulrahman Alobaid

**Affiliations:** 1 Biochemistry Department, KMU Institute of Medical Sciences, Kohat 26000, Pakistan; drfoziazeb@yahoo.com (F.); asmatshaheen@yahoo.com (A.S.); 2 Department of Chemistry, Kohat University of Science & Technology, Kohat 26000, Pakistan; syedbadaramin@gmail.com; 3 Department of Botany, Kohat University of Science & Technology, Kohat 26000, Pakistan; ahmed_kust@yahoo.com; 4 Department of Pharmacognosy (MAPPRC), College of Pharmacy, King Saud University, Riyadh 11451, Saudi Arabia; 5 Department of Pharmaceutical Chemistry, College of Pharmacy, King Saud University, Riyadh 11451, Saudi Arabia; abari@ksu.edu.sa (A.B.); aalobaid1@ksu.edu.sa (A.A.); 6 Department of Soil Science, College of Food and Agriculture Sciences, King Saud University, P.O. Box 2460, Riyadh 11451, Saudi Arabia; msohaib@ksu.edu.sa; 7 Department of Pharmacology, College of Pharmacy, King Saud University, Riyadh 11451, Saudi Arabia; harshad@ksu.edu.sa

**Keywords:** *Ballota pseudodictamnus*, Lamiaceae, phytochemicals, antioxidative and antimicrobial activities

## Abstract

Bioassays guided phytochemical investigations on the ethyl acetate-soluble fraction of the root material of *Ballota pseudodictamnus* (L.) Benth. led to the isolation of two new compounds, ballodiolic acid A (**1**) and ballodiolic acid B (**2**), along with three known compounds ballodiolic acid (**3**), ballotenic acid (**4**), and β-amyrin (**5**), which were also isolated for the first time from this species by using multiple chromatographic techniques. The structures of the compounds (**1**–**5**) were determined by modern spectroscopic analysis including 1D and 2D NMR techniques and chemical studies. In three separate experiments, the isolated compounds (**1**–**5**) demonstrated potent antioxidant scavenging activity, with *IC_50_* values ranging from 07.22–34.10 μM in the hydroxyl radical (^•^OH) inhibitory activity test, 58.10–148.55 μM in the total ROS (reactive oxygen species) inhibitory activity test, and 6.23–69.01 μM in the peroxynitrite (ONOO^−^) scavenging activity test. With *IC_50_* values of (07.22 ± 0.03, 58.10 ± 0.07, 6.23 ± 0.04 μM) for ^•^OH, total ROS, and scavenge ONOO^−^, respectively, ballodiolic acid B (**2**) showed the highest scavenging ability. Antibacterial and antifungal behaviors were also exposed to the pure compounds **1**–**5**. In contrast to compounds **4** and **5**, compounds **1**–**3** were active against all bacterial strains studied, with a good zone of inhibition proving these as a potent antibacterial agent. Similarly, compared to compounds **3**–**5**, compounds **1** and **2 **with a 47 percent and 45 percent respective inhibition zone were found to be more active against tested fungal strains.

## 1. Introduction

A variety of foods have been found to contain antioxidants that scavenge active oxygen species (free radicals) and are generally referred to as scavengers [1]. In order to protect plants that are exposed to sunlight and live under severe oxygen stress, many oxidants are phytochemicals and play an important role. Antioxidants also play an important role in human health, because the biologic protective system cannot work under extreme oxygen stress. According to recent studies, activated oxygen is thought to be a major factor in ageing, hardening of the arteries, diabetes, cancer, and tissue in injury skin [2]. Indeed, approximately 90% of age-related diseases are linked to activated oxygen. In the inflammatory process, cardiovascular disease [3], arteriosclerosis, malaria, rheumatoid arthritis, neurodegenerative disease and the ageing process, and free radicals have important significance [4]. In the etiology of a number of human degenerative diseases, free radicals and ROS or RNS (reactive nitrogen species), including H_2_O_2_, ^•^O^2−^, ^•^OH, NO^•^, and ONOO^−^, play a significant role. As a consequence of aerobic metabolism, these reactive species are produced in the body and harm all intracellular components, such as nucleic acids, proteins, and lipids. ROS are also involved in both ageing and multiple degenerative diseases [3,4].

The genus *Ballota* (Lamiaceae or Mint family) is a medium-sized genus comprising approximately 33–35 species found mainly in the Eurasian and Mediterranean regions [5]. Various members of the genus *Ballota* are widely found in Khyber Pakhtunkhwa and the lower hills of West Punjab in Pakistan. Four species, namely *Ballota psedodictamnus*, *Ballota aucheria*, *Ballota nigra,* and *Ballota limbata* [6] are present in Pakistan. Plants of the genus *Ballota* have been used historically in treatment of burns, wounds, preventing coughs, upper respiratory system inflammation, antispasmodic, neurosedative, diuretic, antiulcer, nausea, antihemorrhoidal, vomiting, and nervous dyspepsia and also are used as antioxidant, antimicrobial, anti-inflammatory, antiemetic, and as hepato-protective [7,8].

Previous phytochemical studies of the *Ballota* genus have led to the isolation of numerous compounds, including diterpenoides such as balloaucherolide, hispanone, 7α-acetoxymarrubiin, ballonigrinone, hispanolone, ballonigrin, and rupestralic acid, and flavonoids like luteolin, salvigenin, nuchensin, ladanein, tangeretin, verbascoside, and apigenin [9,10,11,12,13,14,15,16,17,18,19].

The literature survey showed that no phytochemical research was done on the *Ballota pseudodictamnus* (L.) Benth. plant, which prompted us to conduct phytochemical research on this species. Previous biological evaluation of *Ballota pseudodictamnus* (L.) Benth. by our group confirmed that compared to leaves and stem extracts, the ethyl acetate soluble fraction of root of this plant have more potency against different bacterial and fungal strains [20]. Further, among the all tested solvent soluble fractions, the ethyl acetate soluble fraction of root of the title plant was found to be the most promising for the antioxidant activity and is therefore selected for further phytochemical investigations.

**Figure 1:**
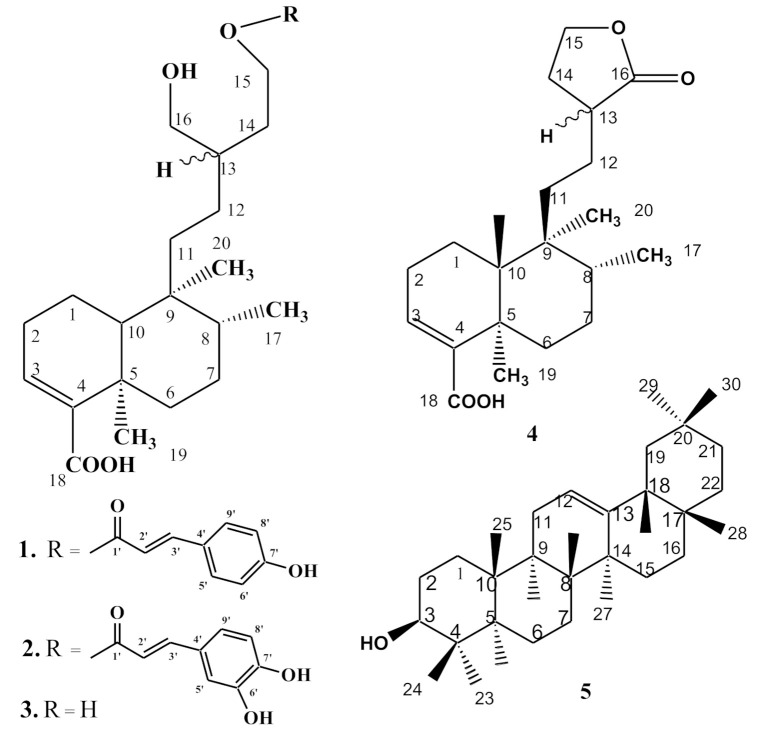
Structures of Compounds **1**–**5**.

Herein in this article, we report the isolation and structural elucidation of two new compounds, ballodiolic acid A (**1**) and ballodiolic acid B (**2**), along with three known phytochemicals ballodiolic acid (**3**), ballotenic acid (**4**), and β-amyrin (**5**), which were isolated for the first time from *Ballota pseudodictamnus* (L.) Benth. and were characterized by various 1D and 2D NMR techniques (Figure 1). The antioxidant activities of the compounds (**1**–**5**) against four different tests along with antibacterial and antifungal activities of the compounds (**1**–**5**) were performed. Though the structures of the compounds (**3**–**5**) were published previously from other plant species, their antimicrobial and antioxidative activities were not.

## 2. Materials and Methods

### 2.1. General Experimental Procedures

To assess the UV spectrum of compounds, (Shimadzu UV-240, Hitachi U-3200, Kyoto, Japan) UV-240 spectrophotometers were used. On IRA-I and JASCO-320-A spectrophotometers, respectively, IR spectra have been reported. On Bruker AM-400, AM-500, and AM-600 spectrometers (Thermo Fisher Scientific, Waltham, MA, USA) with 400, 500, and 600 aspect data systems, the ^1^H-NMR and ^13^C-NMR spectra were observed at 400, 500, and 600 MHz z (instrument used Bruker Biospin, Karlsruhe, Germany). As an internal guide, TMS (tetramethylsilane) was used. The JMS-HX-110 data system spectrometer was used to measure the EI-MS spectra of compounds. The Jasco-DIP-360 digital polarimeter (JASCO, Tokyo, Japan) was used to determine the optical rotation of the compounds. TLC was performed with G-25-UV254 pre-coated silica gel plates, and detection was carried out at 254 nm and 10% H_2_SO_4_ by ceric sulphate. For column chromatography and flash chromatography, respectively, silica gel (E. Merck,70–230 mesh) and silica gel (E. Merck, 230–400 mesh) were used.

### 2.2. Plant Material

*Ballota pseudodictamnus* (L.) Benth. plant sample from Latamber, Karak District, Khyber Pakhtunkhwa (Pakistan) was collected in adequate quantity and was described by Dr. Nisar Ahmad, Assistant Professor, Department of Botany, Kohat University of Science and Technology, Kohat, Pakistan.

### 2.3. Extraction and Isolation of compounds

At room temperature, the shade-dried and grinded root material (04 Kg) of *Ballota pseudodictamnus* (L.) Benth. was exhaustively extracted with methanol. The extract was dried and measured in the (55 g). The crude extract was suspended in water and then was successively partitioned between *n*-hexane (11 g), chloroform (7 g), ethyl acetate (8 g), *n*-butanol (12 g), and aqueous extracts (16 g). Ethyl acetate-soluble fraction of the title plant was found to be the most promising for the said activities and is therefore selected for further phytochemical investigation. The fraction of ethyl acetate was subjected to silica gel chromatography using *n*-hexane with an ethyl acetate gradient of up to 100 percent. Four F1, F2, F3, and F4 sub-fractions were obtained from the compilation process. Furthermore, sub-fraction F1 was subjected to column chromatography and eluted with EtOAc: hexane (1:9) for purification of compound 5 [21]. Similarly, sub-fraction F2 was further referred to column chromatography by eluting the fraction with *n*-hexane-ethyl acetate (6:4). From this fraction, on successive chromatography with *n*-hexane-ethyl acetate (2:8 and 1:9) elution, two pure compounds 2 [22] and 3 [22] were extracted, respectively. The sub-fraction F3 was further subjected to column chromatography by carry-out elution with *n*-hexane-ethyl acetate (5:5). Two pure compounds 1 and 2 were supplied by the fraction. On TLC plates, the purity of the compounds was confirmed.

### 2.4. Ballodiolic Acid A (1)

Light yellow oil (21 mg); molecular formula C_29_H_41_O_6_; molecular weight *m/z* 466.5048 (Calcd 466.6037 for C_29_H_40_O_6_-H_2_O) [M-H_2_O]^+^; IR (KBr) ν_max_: 3634, 2930, 1720, 1715, 1625, 1590 cm^−1^; UV (MeOH) λ_max_: 216 (3.12) nm; [α]_D_^23^ −20.7° (c = 0.155, CHCl_3_).

### 2.5. Ballodiolic Acid B (2)

Pale yellow oil (19 mg); molecular formula C_29_H_41_O_7_; molecular weight *m/z* 482.5544 (Calcd 482.6058 for C_29_H_40_O_7_-H_2_O) [M-H_2_O]^+^; IR (KBr) ν_max_: 3630, 2925, 1722, 1715, 1628, 1591 cm^−1^; UV (MeOH) λ_max_: 216 (3.12) nm; [α]_D_^23^ −20.3° (c = 0.150, CHCl_3_).

### 2.6. Ballodiolic Acid (3)

Colorless oil (17 mg); molecular formula C_20_H_34_O_4_; molecular weight *m/z* 320.2722. (Calcd 320.4618 for C_20_H_34_O_4_-H_2_O) [M-H_2_O]^+^; IR (KBr) ν_max_: 3632, 3590, 2934, 1688 cm^−1^; UV (MeOH) λ_max_: 214 (3.5) nm, [α]_D_^23^ −19.6° (c = 0.170, CHCl_3_) [22].

### 2.7. Ballotenic Acid (4)

Colorless oil (15 mg); molecular formula C_20_H_30_O_4_; molecular weight *m/z* 316.2240 (Calcd 316.4306 for C_20_H_30_O_4_-H_2_O) [M-H_2_O]^+^; IR (CDCl_3_) cm^−1^ 1765, 1675 and 2925; UV (MeOH) λ_max_ 213 nm (3.8); [α]_D_^23^ −0.51° (c = 0.103, CHCl_3_). The ^1^H- and ^13^C-NMR data of the compound were in complete agreement with those reported in literature for ballotenic acid [22].

### 2.8. β-amyrin (5)

Needle-like crystal (25 mg); melting point 197–198 °C; molecular formula C_30_H_50_O; molecular weight *m/z* 426.38; IR (KBr) ν_max_: cm^−1^ 3510, 1636. The ^1^H- and ^13^C-NMR data of the compound was in complete agreement to those reported in literature for *β*-amyrin [21]. 

### 2.9. Alkaline Hydrolysis of Compounds 1 and 2

The Compound **1** (10 mg) in benzene (25 mL) was refluxed with 5% methanolic potassium hydroxide (15 mL) for 4 h, concentrated in vacuo, diluted with water (30 mL), and extracted with ether. The aqueous layer was acidified with dil. hydrochloric acid and extracted with dichloromethane. The residue recovered from the organic phase was characterized as *p*-hydroxy cinnamic acid, colorless crystals from H_2_O, mp 212 °C (characterized through m.m.p., Co-TLC, superimposable IR). Corresponding hydrolysis of **2** afforded 3,4-dihydroxy cinnamic acid (characterized through m.m.p., IR, Co-TLC).

### 2.10. Evaluation of Antioxidative Activity

#### 2.10.1. Measurement of Total ROS Generation Inhibition

Rat kidney homogenates prepared from the kidneys of newly killed male Wistar rats, 130–180 g in weight, were mixed with or without the suspension of extracts or compounds, which were dissolved in 10% EtOH (final concentration: 0.4%). The mixtures were then incubated with 12.5 mM 2’,7’-dichlorodihydrofluorescein diacetate (DCFH-DA, Molecular Probes Inc., Eugene, Oregon), which was dissolved in 100% EtOH (final concentration: 0.2%), at 37 °C for 30 min. A 50 mM phosphate buffer (Wako Pure Chemical Industries, Osaka, Japan) solution at pH 7.4 was also used. DCFH-DA is a stable compound that is hydrolyzed by intracellular esterase to create a reduced, nonfluorescent compound of 2’,7’-dichlorodihydrofluorescein (DCFH). The ROS produced by the homogenates oxidizes the DCFH to a highly fluorescent 2’,7’-dichlorofluorescein (DCF). A microplate fluorescence spectrophotometer (Bio-Tek Instruments Inc., Winooski, VT) with excitation and emission wavelengths of 460 and 530 nm, respectively, was used to track the fluorescence intensity of the oxidized DCF [23]. As a positive regulation, Trolox has been used as an important standard oxidant.

#### 2.10.2. Measurement of Hydroxyl Radical Generation inhibition

First, 1 mM H_2_O_2_ and 0.2 mM FeSO_4_ (Fisher Science, Fair Lawn, NJ, USA) were applied to extracts or compounds dissolved in 10 percent EtOH (final concentration: 0.4 percent) and incubated at 37 °C for 5 min. Then, 2 M DCFH-DA (Molecular Probes Inc., Eugene, Oregon) treated with esterase in 100 percent EtOH was introduced, and fluorescence changes were tracked on a microplate fluorescence spectrophotometer (BioTek Instruments Inc., Winooski, VT, USA) with 460 and 530 nm excitation and emission wavelengths, respectively, for 30 min [24]. As a positive regulation, Trolox, an efficient standard oxidant, was used.

#### 2.10.3. Measurement of ONOO^−^ Scavenging Activity

The ONOO^−^ scavenging activity was calculated by monitoring the oxidation of dihydrorhodamine 123 (DHR 123, Molecular Probes Inc., Eugene, OR, USA) using a slight modification of the reported method [25]. At 80 °C, DHR 123 (5 mM) in DMF, purged with N_2_, was stored as a stock solution. Prior to the analysis, this solution was then put on ice and held in the dark. The buffer consisted of 90 mM NaCl, 50 mM Na_3_PO_4_, 5 mM KCl at pH 7.4, and 100 M diethylenetriaminepentaacetic acid (DTPA), each of which was prepared and purged with N_2_ with high quality deionized H2O.The final DHR 123 concentration amounted to 5 M. The context and final fluorescent intensities were assessed with and without the authentic ONOO^−^ 5 min after treatment. The authentic ONOO^−^oxidized DHR 123 was rapidly oxidized, and the final fluorescent strength of the oxidized DHR 123 was determined using the FL 500 microplate fluorescence reader (BioTek Instruments Inc., Winooski, VT, USA) at 480 and 530 nm excitation and emission wavelengths, respectively. For the final fluorescence intensity minus background fluorescence, the results are expressed as the mean ± standard error (*n* = 3). The results are expressed as DHR 123 oxidation percent inhibition, and DLpenicillamine, the normal oxidant, has been used as a positive regulation. In all three scavenging experiments, the IC_50_ was classified as sample concentrations showing 50 percent scavenging activity and was calculated from a triplicate experiment.

### 2.11. Antimicrobial Screening of Compounds **1**–**5**

#### 2.11.1. Stock Solution Preparation

First, 30 μg of each compound was dissolved in 1mL of dimethyl sulfoxide oxide (DMSO) to form a solution of 30 μg/mL concentration in the preparation of the stock solution. For the present analysis, DMSO was chosen as a solvent, because it shows no activity against bacteria [26].

#### 2.11.2. Anti-Bacterial Assay

To test the antibacterial activities of isolated compounds, the process of Agar diffusion was used. In the antibacterial bioassay, five bacterial strains were used, i.e., *Escherichia coli*, *Pseudomonas aeruginosa, Salmonella typhi, Bacillus subtilis,* and *Staphylococcus aureus*. The various bacterial strains were sub-cultivated for a second time to obtain new and fresh culture of each strain in order to test the antibacterial activities of acquired compounds. The nutrient broth was inoculated with a single colony of each strain, then it was incubated for 24 h at 37 °C. Nineteen grams of nutrient agar was dissolved in 1L of distilled water, and the resulting solution was autoclaved for 30 min at 121 °C. Then, 75 mL of solid media was prepared after cooling by pouring the media into Petri dishes (14 cm). Four holes per plate were made with sterile cork borer in these media (8 mm). For inoculation of all bacterial strains, a single Petri dish was used. DMSO (dimethyl sulfoxide) was prepared in the stock solution of compounds 1–3. Two solutions of DMSO (negative control) and that of levofloxacin (positive control) were used in these specific holes for 100 μL of each solution. The activity of each hole zone was calculated in mm when the generic drug inhibition zone (Levofloxacin) was compared using the protocol [26].

#### 2.11.3. Anti-Fungal Assay

For the determination of antifungal activities of isolated compounds, the Disk diffusion method was used. In an antifungal bioassay of isolated compounds, five fungal strains were used: *Aspergillus flavus*, *Fusarium solani*, *Aspergillus fumigatus*, *Aspergillus niger,* and *Canadida glabrata*. The old fungal strains were subcultivated using nutrient broth medium in order to evaluate the antifungal activities of the obtained compounds and to obtain the fresh culture of each fungal strain. For seven days at 28 °C, the new cultures were incubated. Every fungal strain was inculcated through point inoculation on a separate potato dextrose agar plate (PDA). Then, 100 μL of stock solution of compounds 1–3, pure DMSO (negative control) and Miconazole (positive control) were used for experiments. After incubation for seven days, the activity of each compound was measured at a temperature of 28 °C [27].

## 3. Results and Discussion

The ballodiolic acid A (**1**) was isolated as light yellow oil with molecular formula of C_29_H_40_O_6_, which was confirmed through HR-EIMS having molecular ion peak at *m/z* 466.5048 (Calcd 466.6037 for C_29_H_40_O_6_-H_2_O) accompanied by loss of water molecule [M-H_2_O]^+^. The IR spectrum for the compound **1** at 3630 cm^−1^ indicates the presence of free hydroxyl group (OH); α,β-unsaturated carbonyls were observed at 1715 and 1689 cm^−1^. The IR spectrum revealed the presence of the aromatic group 1590 cm^-1^ and broad IR band at 2925 cm^−1^ confirm the presence hydroxyl (OH) moiety of α,β-unsaturated carboxylic acid. For the compound **1**, three methyl protons signals (Table 1) were observed in ^1^H NMR spectrum at *δ* 0.77 (3H, d, J = 5.1 Hz, H-17), *δ* 1.3 (3H, s, H-19), and *δ* 0.73 (3H, s, H-20); an olefinic proton at *δ* 6.82 (1H, br s, H-3); the oxygenated methylene protons appeared at *δ* 3.69–4.1 (2H, m, H-15); and free hydroxyl methylene protons appeared at and *δ* 3.75–3.79 (2H, m, H-16), respectively. The other protons signals appeared at *δ* 0.79–0.88 (2H, m, H-1), *δ* 2.17–2.37 (2H, m, H-2), *δ* 2.39–2.40 (2H, m, H-6), *δ* 1.38–1.50 (2H, m, H-7), *δ* 1.44 (1H, m, H-8), *δ* 1.38 (1H, d, J = 10.3 Hz, H-10), *δ* 1.35–1.45 (2H, m, H-11), *δ* 1.63–1.68 (2H, m, H-12), *δ* 1.55 (1H, m, H-13), and *δ* 1.20–1.27 (2H, m, H-14), suggesting that compound **1** has bicyclical clerodane diterpene skeleton [22]. The ^13^C NMR spectral analysis of compound **1** (Table 1) also confirmed bicyclical clerodane diterpene skeleton on the basis of distortionless enhancement by polarization transfer (DEPT) and broad band (BB) NMR experiments. The carboxylic group carbon and two oxygenated methylene carbon resonated at *δ* 172.7, 62.7, and 61.8 for C-18, C-15, and C-16, respectively. The olefinic carbons shows signals at *δ*141.0 for C-3 and at *δ* 141.9 for C-4. The signals for the two tertiary methyl and for one secondary methyl group observed at C-19 *δ* 20.6, C-20 *δ* 18.7, and C-17 *δ* 16.3. For seven upfield methylene carbons and four methines carbons, signals resonating at *δ* 17.9, 27.7, 37.2, 35.3, 27.5, 36.4, 38.5, 46.8, 35.5, 24.8, 39.9, and 29.9 were assigned to C-1, C-2, C-5, C-6 C-7, C-8, C-9, C-10, C-11, C-12, C-l3, and C-14, respectively. The above spectral data were closely related to (ballodiolic acid **3**) (Table 1) [22], differing in having additional characteristic signals for *p*-hydroxycinnemoyl moiety (aromatic protons showing AA‘, BB‘ pattern with *δ* 7.40 (2H, d, *J* = 8.1 Hz) and *δ* 6.87 (2H, d, *J* = 8.1 Hz); trans olefinic protons at *δ* 7.55 (1H, d, *J* = 16.3 Hz) and *δ* 6.47 (1H, d, *J* = 16.3 Hz)), and the downfield signals in ^13^C NMR spectra at *δ* 166.3 and 117, 144.7 could be assigned to the cinnamoyl carbonyl group and its olefinic carbons, respectively (Table 1). The presence of *p*-hydroxycinnemoyl moiety was further confirmed by electron impact mass spectrum (EI-MS) showing intense peaks at *m/z* 147.

**Figure 2:**
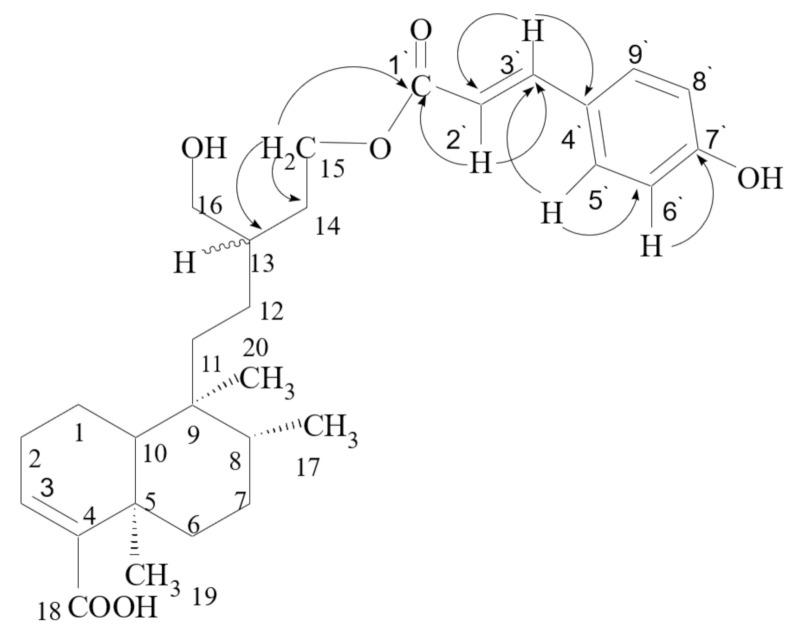
Important HMBC Correlations of **1.**

All the assignments were further confirmed by ^1^H-^13^C multiple bond interaction in heteronuclear multiple-quantum coherence (HMBC) spectrum of **1** (Figure 2). Beside the ^1^H-^13^C multiple bond correlations established earlier for ballodiolic acid [22], additional interactions were observed by the oxymethylene protons (H-15 protons), which showed ^2^*J* and ^3^*J* correlations with C-14 (*δ* 36.4) and C-13 (δ 39.9), respectively, and are also correlated with carbonyl carbon of cinnamoyl moiety (*δ* 166.3). The olefinic proton (H-2′) at δ 6.47 of *p*-hydroxycinnemoyl moiety showed cross peak with carbonyl carbon (*δ* 166.3) and C-3′ (*δ* 144.7). These correlations of HMBC spectrum confirmed the attachment of cinnamoyloxy methylene at C-14. All these evidences were in accordance to the assigned structure of (*E*)-5-(3-(hydroxymethyl)-5-(3-(4-hydroxyphenyl)acryloyloxy)pentyl)-5,6,8a-trimethyl-3,4,4a,5,6,7,8,8a-octahydronaphthalene-1-carboxylic acid as ballodiolic acid A (**1**).

Ballodiolic acid B (**2**) was obtained as a pale yellow oil, molecular formula C_29_H_40_O_7_ by [M]^+^ peak at *m/z* 482.5544 in HR-EIMS spectrometry (calcd. for C_29_H_40_O_7_; 482.6058). The UV, IR, ^1^H, and ^13^C NMR spectra of **2** were almost identical to those of **1**, suggesting that **2** had the same skeleton as that of **1**, except the difference due to 6′,7′-dihydroxylcinnamoyl moiety, which was confirmed by ^1^H NMR (ABX system at *δ* 7.15 (1H, d, *J* = 2.3 Hz, H-5′), *δ* 7.07 (1H, dd, *J* = 2.3, 8.1 Hz, H-9′), and *δ* 6.75 (1H, d, *J* = 8.1 Hz, H-8′)) [28]. The ^13^C NMR spectra (Table 1) of **2** also confirmed the 6′,7′-dihydroxylcinnamoyl group showing downfield signals at *δ* 150.3 (C-7′) and *δ* 148.2 (C-6′) [28] and was further confirmed by electron impact mass spectrum (EI-MS) showing intense peaks at *m/z* 163. All the above spectral data confirmed the structure of compound 2 as (*E*)-5-(5-(3-(3,4-dihydroxyphenyl)acryloyloxy)-3-(hydroxymethyl)pentyl)-5,6,8a-trimethyl-3,4,4a,5,6,7,8,8a-octahydronaphthalene-1-carboxylic acid (**2**).

Alkaline hydrolysis of **1** and **2** provided *p*-hydroxy cinnamic acid and 3,4-dihydroxy cinnamic acid, respectively (characterized through m.m.p., Co-TLC, superimposable IR), providing conclusive evidence for the presence of these moieties in the respective compounds. The important HMBC correlations were in complete agreement to the assigned structures of compounds **1** and **2**.

For the isolated compounds **1**–**5** and different solvent soluble extracts, we investigated the general antioxidant effects of the compounds and extracts to inhibit OH and absolute ROS and to scavenge genuine ONOO^−^. The different solvent soluble fractions of root, stem, and leave parts of *Ballota* *pseudodictamnus* exhibited remarkable anti-oxidant activities (Table 2). The ethyl acetate soluble fraction of root part of the plant showed stronger anti-oxidative activity than that of the other fractions in all the three scavenging tests with the IC_50_ values of 19.10 ± 0.05, 65.13 ± 0.05, and 20.18 ± 0.05 for ^•^OH, ROS, and ONOO^−^, respectively. The chloroform soluble fraction of root part of the plant observed the ^•^OH, ROS, and ONOO^−^ scavenging activities with IC_50_ values of 47.28 ± 0.07, 80.19 ± 0.03, and 41.23 ± 0.07 µg/mL, respectively (Table 2).

In those three studies, Compounds **2**, with one aliphatic hydroxyl, one carboxylic acid, and 3,4-dihydroxy cinnamoyl moieties and **1,** with 3-hydroxy cinnamoyl, one aliphatic hydroxyl, one carboxylic acid moieties, showed greater potential to scavenge ^•^OH, total ROS, and ONOO^−^ with the IC_50_ values (07.22 ± 0.03, 58.10 ± 0.07, and 6.23 ± 0.04 μM and 09.15 ± 0.07, 64.09 ± 0.02, and 8.16 ± 0.01 μM), respectively. Compound **3** with two free hydroxyl groups and carboxylic acid moiety in its structure showed good antioxidant activity (Table 2). Lactone and carboxylic acid moiety compounds **4** displayed moderate activity with IC_50_ values (19.08 ± 0.05, 69.15 ± 0.08, and 21.13 ± 0.09 μM) for ^•^OH, total ROS, and ONOO^−^, respectively, for scavenge. *β*-amyrin (**5**) with one hydroxyl moiety showed lower activity, but still significant activity in all the scavenging activity tests (Table 3).

The antioxidative activity was also examined in terms of the chemical structures including those of functional radical and its orientation. Hydroxyl groups in the 1,4- and 1,3-orientation at cinnamoyle are mainly involved in the scavenging potential of compounds **1** and **2**. Based on these results, a benzene ring where the hydroxyl radical is in 1,4-orientation allow the oxygen atom to share a positive charge, thereby causing stabilization through delocalization and hence increasing the antioxidant activity as was observed in compound **1**. Because of the electron donating effect of the hydroxyl group in 1,3-orientation, it helps to stabilize positive charge, and this is taught to influence the scavenging ability. This was observed in case of compound **2** where further enhanced scavenging activity was observed as this compound bear 3,4-dihydroxy cinnamoyl moieties.

The antibacterial and antifungal activities of compounds (**1**–**5**) against the various bacterial strains *Escherichia coli*, *Pseudomonas aeruginosa*, *Salmonella typhi*, *Bacillus subtilis*, and *Staphylococcus aureus* (Table 4) were observed in this study. Table 4, displays the compounds (**1**–**5**) from the test samples in terms of diameters of inhibition zones. Against the examined microorganisms, such as *Escherichia coli*, *Salmonella typhi,* and *Staphylococcus aureus*, the compounds were found to be prominently active. In contrast to compound **3**–**5**, compounds **1** and **2** were active against all bacterial strains studied, with an inhibition zone of 12 ± 0.03 and 11 ± 0.01 mm, respectively. In the same concentration range, compound 3 demonstrated good antibacterial activity against the test species. Antifungal activity of these compounds (**1**–**5**) against *Aspergillus flavus*, *Fusarium solani*, *Aspergillus fumigatus*, *Aspergillus niger,* and *Canadida glabrata*, as shown in Table 5, with an inhibition zone of 7% to 47%. Compound **2** showed the highest antifungal activity with a 47% to 25% inhibition zone, while the other two compounds **1** and **3** showed good antifungal activity against the fungal strains listed above. In recent years, while technology and medicine have grown widely, due to a reduction in natural wealth and disadvantages, some countries have found it necessary to use natural products for several different purposes. As in several other nations, plants known to individuals with health benefits are collected and used in Pakistan for the treatment of various diseases. The antimicrobial and antifungal capabilities of compounds (**1**–**5**) isolated from *Ballota pseudodictamnus* have been identified in this study. Owing to the strength of widespread resistance to them, the use of certain antibiotics is no longer recommended. As shown in Table 4, these compounds can also be used instead of antibiotics; they have shown visible antibacterial activity against *Escherichia coli*, *Pseudomonas aeruginosa*, *Salmonella typhi*, *Bacillus subtilis*, and *Staphylococcus aureus*. In addition, against different strains such as *Aspergillus flavus*, *Fusarium solani*, *Aspergillus fumigatus*, *Aspergillus niger,* and *Canadida glabrata*, the antifungal activity of these compounds was prominent (Table 5).

The established antimicrobial mechanisms associated with each group of chemicals to which the isolated compounds belong can explain the antimicrobial potential of *Ballota pseudodictamnus* solvent soluble fractions and compounds **1**–**5**. Disruption of the membrane has been proposed as one of the possible mechanisms of action [29,30]. The antimicrobial activities of compounds **1**–**5** may also be clarified by this. The bacterial and fungal organisms differed in their antimicrobial activities. The genetic differences between the microorganisms [29,30] may be the explanation for these variations. This is interesting in view of the possibility of new antimicrobial medicines being produced from natural products.

Based on structure–activity relationships, the hydroxyl substitution pattern on benzene ring is an indicator of antimicrobial activity for compounds, and the additional hydroxyl group at 4̕-position significantly increases the activity, while the methylation of these hydroxyl groups reduces the antibacterial potential at different levels. Besides this, the addition of the 3-hydroxyl group seems to enhance the activity [31,32,33]. Here in our reported compounds, **2** and **3** have 3-hydroxyl group and 3,4-dihydroxyl substitution pattern, respectively, and hence the compound **3** was found more active than compound **2**, followed by compounds **1**, **4,** and **5** (Table 4 and Table 5).

Similarly, the antioxidant properties of the *Ballota* species growing in Turkey were investigated by Citoğlu GS et al. The authors recorded the antioxidant properties of ethanol extracts for superoxide anion formation and lipid peroxidation of 16 species of *Ballota* cultivated in Turkey. The extracts of *Ballota glandulosissima*, *Ballota antalyense*, *Ballota larendana*, *Ballota pseudodictamnus*, *Ballota macrodonta*, *Ballota nigra* subsp. *anatolica*, *Ballota rotundifolia*, *saxatilis*, *Ballota saxatilis* subsp., and *Ballota saxatilis* subsp. *brachyodonta* with IC_50_ values of 0.50 to 0.87 mg/mL exhibited remarkable anti-superoxide anion formation [34].

The in vitro antioxidant function of ethanol extracts extracted from 21 aromatic plants belonging to the family Lamiaceae has been investigated by Couladis et al. *Salvia ringens*, *Stachys spruneri*, *Phlomis lanata*, *Salvia pomifera*, *Origanum dictamnus*, *Ballota pseudodictamnus*, *Ballota acetabulosa*, *Teucrium polium*, *Calamintha glandulosa,* and *Micromeria graeca* exhibited the same behavior as α-tocopherol among the extracts tested [35]. The authors also studied the chemical composition of the *Ballota pseudodictamnus* essential oil obtained by GC/MS from the aerial components. Aryophyllene oxide, phytol, and γ-muurolene were the major components of the 52 reported constituents of the crude. In addition, the essential oil for its antimicrobial activity has been investigated [36].

Previously, our research group reported in vitro antimicrobial, antiprotozoal activities and heavy metals toxicity of different parts of *Ballota pseudodictamnus* (L.) Benth. [20]. The analysis was conducted to verify the function of different sections of *Ballota pseudodictamnus* (L.) Benth. in antimicrobial and antiprotozoal terms. These behaviors were then compared with the toxicity of heavy metals in various parts, plants accumulating in different amounts in different parts. Ethanolic extract, chloroform, and ethyl acetate fractions in the *Ballota pseudodictamnus* (L.) Benth. roots showed antileishmanial activity in in-vitro antileishmanial results. The fraction of ethanol, *n*-butanol, and ethyl acetate in the stem showed inhibition of leishmania amastigote type. Inhibition of the leishmanial parasite was seen in the ethanol extract, chloroform, and *n*-butanol fraction in the leaves. Results have shown that different parts of the plant have different properties of inhibition [20].

Diterpenes and diterpenoids have antioxidant activity. Carnosol and carnosic acid are inhibitors of lipid peroxidation; they prevent the oxidation of fatty acids, triglycerides, and low-density lipoproteins in human aortic endothelial cells [37,38]. Under oxidative stress, nematodes treated with carnosol had a 21% increase in lifespan compared to controls, and under heat stress increased worm survival was higher by 9% [39]. The combined action of carnosic acid and carnosol against ROS and lipid radicals makes this diterpenoid tandem an effective antioxidant defense. In the Ames test, carnosol was found to have significant antioxidant and anti-mutagenic activity comparable to ascorbic acid [40]. In a micronucleus test, it was found that carnosol is even more effective than ascorbic acid in protection against gamma radiation [41]. Carnosol protects cells from eco-toxicants [42].

Diterpenes and diterpenoids are reported for antiviral, antibacterial, antiparasitic, antifungal, and antiprotozoal action. Some terpenoids are toxic to microorganisms and insects and play an important role in plant protection [43,44]. Extract of *Stevia* and its glycosides (for example, steviol), in addition to their value as sweeteners, has a therapeutic effect against cystic fibrosis. Carnosic acid and carnosol, isosteviol, andrographolide, and dehydroabietic acid have several important protective properties, including anti-tuberculosis and antiseptic, and can be used in the treatment of colds, showing antibacterial and antiviral activity [45,46].

Kadidiatou O. et al. investigated *Plectranthus madagascariensis*-isolated antimycobacterial, cytotoxic, and antioxidant activities of abietane diterpenoids. *P. madagascariensis*’ phytochemical study has resulted in the isolation of five recognized royleanone abietanes, namely 6-β,7- alpha-dihydroxyroyleanone, 7 -alpha-acetoxy-6β-hydroxyroyleanone, horminone, coleon U quinone, and carnosolone. The first four compounds demonstrated antimycobacterial activity against *Mycobacterium tuberculosis* H_37_Rv (minimum inhibitory concentration for 90 percent inhibition, MIC_90_ = 5.61–179.60 μM). All the isolated compounds showed antioxidant activity through single-electron transfer (SET) and/or hydrogen-atom transfer (HAT) with compound carnosolon being the most active antioxidant agent [47].

González-Cofrade et al. identified 19 diterpenoids with anti-inflammatory activity. For their anti-inflammatory activities against NLRP3 inflammasome activation, the authors assessed a series of dehydrohispanolone derivatives. IL-1β secretion was substantially inhibited by four derivatives, being the most active (IC50 = 18.7 and 13.8 μM, respectively). Analysis of the expression of IL-1β and caspase-1 showed that these four diterpenoids were selective inflammasome NLRP3 inhibitors, confirming the anti-inflammatory properties of hispanolone derivatives shown earlier [48].

S. Núñez et al. have documented antibacterial activity against three bacteria: *Staphylococcus aureus*, *Escherichia coli,* and *Mycobacterium smegmatis*, two natural diterpenoids isolated from *Azorella* compacta together with six semisynthetic derivatives. There was no antibacterial activity for the natural diterpenoids, mulinolic acid and azorellanol, but six derivatives, 12-oxo-11,13-alpha, alpha-dihydroxymulin-20-oic acid, 11-oxo-12,13-alpha, alpha-dihydroxymulin-20-oic acid, 11,12-dioxo-13-alpha-hydroxymulin-20-oic acid, 7-acetoxymulin-9,12-diene, mulin-9,12-dien-7-ol, and 7-acetoxy-12,13-dihydroxymulin-9-en, were found to be active against three bacteria tested. They indicated that the antibacterial activity reported for six semi-synthetic diterpenoids may not be comparable to that reported for positive control ampicillin, but showed that the biological activity of diterpenoids may be modified [49].

The efficacy of seven predominant wine terpenoids (i.e., alpha-pinene, limonene, myrcene, geraniol, linalool, nerol, and terpineol) against foodborne pathogenic bacteria was determined by Chung-Yi Wang et al., and their antioxidant activities were observed. Against foodborne pathogenic bacteria, antibacterial activities were observed. The MIC_50_ and MBC values were 0.420–1.598 and 0.673–3.432 mg/mL for *Escherichia coli*, *Salmonella enterica*, and *Staphylococcus aureus*, respectively. The fastest free radical scavenging DPPH (IC50 value = 12.57 ± 0.18 mg/mL) and the highest reducing strength (L-ascorbic acid equivalent 213.7 ± 5.27 μg/mL) were shown by the terpenoid alpha-pinene. New possible sources of natural antibacterial and antioxidant agents for use in the food industry may be the seven predominant terpenoids in wines that were found in the recorded study [50].

Antibacterial and antioxidant cassane diterpenoids from *Caesalpinia benthamiana* have been reported by Rita Akosua Dickson et al. Using the microdilution assay method and the DPPH spectrophotometric and TBA lipid peroxidation assays, the antibacterial and antioxidant activities of the isolated cassane diterpenoids were evaluated. For both *Staphylococcus aureus* and *Micrococcus flavus*, Benthaminin 1 was identified as the more active antibacterial compound with MIC values of 47.8 microM. The more active antioxidant compound was found to be benthaminin 2 and showed IC_50_ values of 42.7 microM and 74.2 microM for DPPH and TBA assays, respectively. Deoxycaesaldekarin C possessed both antibacterial and antioxidant activities [51].

Diterpeniod, abietane 7 alpha-acetoxy-6β-hydroxyroyleanone (AHR), obtained from plant extracts, has been stated to be an attractive lead for drug production due to its established antimicrobial properties by Carlos E et al. [52].

The compliance of the biological effects of terpenoids with the parameters of geroprotectors, including primary criteria, was evaluated by Ekaterina Proshkina et al. Among the different terpenoid groups, the number of substances showing the greatest compliance with both primary and secondary geroprotector criteria was found. Terpenoids are therefore an underrated source of potential geroprotectors that can have an effective impact on ageing and age-related disease mechanisms [53].

## 4. Conclusions

The bioassays guided isolation from ethyl acetate soluble fraction of the root material of the plant resulted two new compounds (**1**, **2**) along with three already reported compounds (**3**–**5**). The structures of the compounds **1**–**5** were elucidated with the help of 1D and 2D NMR techniques. The compounds **1**–**5** were found active against different microorganisms and exhibited antioxidative properties. The medicinal efficacy of these compounds will, however, be explored through more in vivo studies.

## Figures and Tables

**Table 1:** ^1^H- (400 MHz) and ^13^C-NMR (100 MHz) assignments (*δ*/ppm) of Compounds 1–3 in CDCl_3_.

Position	1		2		3	
	δ(1H)	δ(13C)	δ (1H)	δ(13C)	δ(1H)	δ(13C)[M1]
1	0.79–0.88 (m)	17.9	0.81–0.90 (m)	17.6	0.80–0.89 (m)	17.5
2	2.17–2.37 (m)	27.7	2.15–2.33 (m)	27.5	2.14–2.34 (m)	27.4
3	6.82 (br s)	141.0	6.81 (br s)	140.8	6.79 (br s)	140.0
4	-	141.9	-	141.5	-	141.3
5	-	37.2	-	37.4	-	37.5
6	2.39–2.40 (m)	35.3	2.35–2.38 (m)	35.5	2.35–2.38 (m)	35.5
7	1.38–1.50 (m)	27.5	1.36–1.47 (m)	27.3	1.38–1.49 (m)	27.2
8	1.44 (m)	36.4	1.45 (m)	36.3	1.46 (m)	36.1
9	-	38.5	-	38.4	-	38.6
10	1.38 (d, 10.3)	46.8	1.35 (d, 10.3)	46.6	1.36 (d, 10.3)	46.6
11	1.35–1.45 (m)	35.5	1.37–1.43 (m)	35.7	1.36–1.44 (m)	35.8
12	1.63–1.68 (m)	24.8	1.62–1.70 (m)	24.7	1.61–1.69 (m)	24.9
13	1.55 (m)	39.9	1.56 (m)	39.5	1.58 (m)	39.8
14	1.20–1.27 (m)	29.9	1.22–1.28 (m)	29.6	1.23–1.29 (m)	29.7
15	3.69–4.1 (m)	62.7	3.67–4.1 (m)	62.3	3.65–4.0 (m)	66.3
16	3.75–3.79 (m)	61.8	3.73–3.78 (m)	61.5	3.70–3.77 (m)	61.1
17	0.77 (d, 5.1)	16.3	0.78 (d, 5.3)	16.1	0.76 (d, 5.3)	15.9
18	-	172.7	-	172.5	-	172.0
19	1.23 (s)	20.6	1.24 (s)	20.4	1.21 (s)	20.5
20	0.73 (s)	18.7	0.71 (s)	18.5	0.70 (s)	18.4
1′	-	166.3	-	165.9	-	-
2′	6.47 (d, 16.3)	117.0	6.45 (d, 16.2)	117.3	-	-
3′	7.55 (d, 16.3)	144.7	7.55 (d, 16.2)	144.9	-	-
4′	-	125.9	-	126.4	-	-
5′	7.40 (d, 8.1)	130.2	7.15 (d, 2.3)	112.9	-	-
6′	6.87 (d, 8.1)	116.0	-	148.2	-	-
7′	-	159.3	-	150.3	-	-
8′	6.87 (d, 8.1)	116.0	6.75 (d, 8.1)	116.0	-	-
9′	7.40 (d, 8.1)	130.2	7.07 (dd, 8.1, 2.3)	124.1	-	-

Multiplicities and coupling constants (*J* = Hz) are given in parenthesis. *δ* in ppm from TMS.

**Table 2:** Antioxidant activity of the solvent fractions of root, stem, and leaves of *B. pseudodictamnus.*

Sample	Solvent Fractions	*IC*_50_ [µg/mL] ^a^		
		^•^OH ^b^	Total ROS ^c^	ONOO^−^^d^
	*n*-hexane	79.17 ± 0.05	>300	80.37 ± 0.03
Root	CHCl_3_	47.28 ± 0.07	80.19 ± 0.03	41.23 ± 0.07
	EtOAc	19.10 ± 0.05	65.13 ± 0.05	20.18 ± 0.05
	*n*-BuOH	31.51 ± 0.03	90.41 ± 0.06	61.16 ± 0.04
	H_2_O	97.37 ± 0.05	>400	89.12 ± 0.05
Stem	*n*-hexane	83.41 ± 0.03	>400	78.25 ± 0.05
	CHCl_3_	55.13 ± 0.05	87.21 ± 0.01	50.47 ± 0.06
	EtOAc	29.15 ± 0.06	75.09 ± 0.05	26.07 ± 0.03
	*n*-BuOH	40.09 ± 0.01	99.34 ± 0.03	60.49 ± 0.05
	H_2_O	91.12 ± 0.05	>400	87.17 ± 0.06
Leaves	*n*-hexane	77.39 ± 0.06	>400	85.13 ± 0.07
	CHCl_3_	59.25 ± 0.03	89.45 ± 0.06	52.26 ± 0.05
	EtOAc	31.15 ± 0.05	73.27 ± 0.03	30.18 ± 0.01
	*n*-BuOH	45.20 ± 0.01	99.19 ± 0.05	67.38 ± 0.07
	Trolox ^e^	6.30 ± 0.03	40.05 ±0.07	-
	DL-Penicillamine ^f^	-	-	2.07 ± 0.09

^a^ Values of OH, total ROS, and ONOO^−^ are expressed as the mean ± standard error of triplicate experiments. ^b^ Inhibitory activity of hydroxyl radical generation in 1.0 mM H_2_O_2_ and 0.2 mM FeSO_4_.^c^ Inhibitory activity of total ROS generation in kidney post microsomal fraction. ^d^ Inhibitory activity of authentic peroxynitrite. ^e^ Trolox was used as a positive control. ^f^ DL-Penicillamine was used as a positive control.

**Table 3:** Antioxidative activity of Compounds 1-5.

**Compound**	**IC** **_50_** **[µM] ^a^**		
	**^•^** **OH ^b^**	**Total ROS ^c^**	**ONOO^− d^**
**1**	09.15 ± 0.07	64.09 ± 0.02	8.16 ± 0.01
**2**	07.22 ± 0.03	58.10 ± 0.07	6.23 ± 0.04
**3**	12.17 ± 0.04	69.15 ± 0.08	10.27 ± 0.02
**4**	19.08 ± 0.05	87.91 ± 0.04	21.13 ± 0.09
**5**	34.10 ± 0.07	148.55 ± 0.05	69.01 ± 0.05
Trolox ^e^	2.51 ±0.03	35.06 ± 0.07	-
DL-Penicillamine ^f^	-	-	1.09 ± 0.07

^a^ Values of OH, total ROS, and ONOO^−^ are expressed as the mean ± standard error of triplicate experiments. ^b^ Inhibitory activity of hydroxyl radical generation in 1.0 mM H_2_O_2_ and 0.2 mM FeSO_4_. ^c^ Inhibitory activity of total ROS generation in kidney post microsomal fraction. ^d^ Inhibitory activity of authentic peroxynitrite. ^e^ Trolox was used as a positive control. ^f^ DL-Penicillamine was used as a positive control.

**Table 4:** Antibacterial activities of compounds **1**–**5** (30 µg/mL) against different bacteria strains.

Name of Bacterial Strain	Zone of Inhibition (mm)					
	Compound 1	Compound 2	Compound 3	Compound 4	Compound 5	Standard Drug Levofloxacin
*E. coli*	11 ± 0.01	12 ± 0.03	10 ± 0.01	8 ± 0.09	7 ± 0.02	16 ± 0.01
*P. aeruginosa*	8 ± 0.02	9 ± 0.02	7 ± 0.08	5 ± 0.06	0 ± 0.01	19 ± 0.02
*S. typhi*	13 ± 0.09	11 ± 0.04	9 ± 0.06	2 ± 0.04	6 ± 0.06	24 ± 0.03
*B. subtilis*	5 ± 0.053	7 ± 0.02	4 ± 0.03	2 ± 0.05	3 ± 0.04	22 ± 0.02
*S. aureus*	7 ± 0.02	9 ± 0.01	6 ± 0.01	3 ± 0.02	5 ± 0.03	20 ± 0.01

Mean ± standard error of triplicate experiments.

**Table 5:** Antifungal activities of compounds **1**–**5** (30 µg/mL) against different fungal strains.

Name of Fungal Strain	Zone of Inhibition (%)					
	Compound 1	Compound 2	Compound 3	Compound 4	Compound 5	Standard Drug Miconazole
*A. flavus*	45%	47%	35%	22%	25%	100%
*F. solani*	30%	34%	27%	9%	15%	100%
*A. fumigatus*	33%	39%	30%	11%	17%	100%
*A. nigar*	21%	25%	17%	15%	11%	100%
*C. glabrata*	25%	29%	22%	17%	7%	100%

## Data Availability

All available data incorporated in the manuscript.
